# Potential for *Acanthoscelides obtectus* to Adapt to New Hosts Seen in Laboratory Selection Experiments

**DOI:** 10.3390/insects10060153

**Published:** 2019-05-29

**Authors:** Uroš Savković, Mirko Đorđević, Biljana Stojković

**Affiliations:** 1 Department of Evolutionary Biology, Institute for Biological Research “Siniša Stanković”, University of Belgrade, Bulevar despota Stefana 142, 11060 Belgrade, Serbia; mirko.djordjevic@ibiss.bg.ac.rs (M.Đ.); bilja@bio.bg.ac.rs (B.S.); 2 Institute of Zoology, Faculty of Biology, University of Belgrade, Studentski trg 16, 11000 Belgrade, Serbia

**Keywords:** bruchids, stored product commodities, population dynamics, host shift, experimental evolution, seed beetle, *Acanthoscelides obtectus*

## Abstract

Effective pest management strategies for a targeted pest species must rely on accurate, reliable and reproducible estimates of population dynamics. Importance of such approaches is even more conspicuous when assessing pest’s potential to utilize other stored products. Using an experimental evolution approach, we have focused our attention on a common bean pest, the seed beetle (*Acanthoscelides obtectus*). We looked into the potential to invade and sustain population growth on two suboptimal host plants (chickpeas and mung beans). Such an approach simulates steps of the host-shift process in storages. By analyzing population dynamics during initial encountering with a new host plant, we detected a population drop for both novel hosts. However, transgenerational development in a novel environment resulted in a constant population growth in chickpeas, but not in mung bean populations. Reversal of chickpea selected populations to original host plant has led to a severe decrease in population parameters due to low viability of immatures, while the opposite trend was detected in mung bean populations. This paper highlights the importance of good practice in estimating population dynamics for economically important species. With special emphasis on storage pest species, we discuss how this approach can be useful for estimating invading potential of pest insects.

## 1. Introduction

More than 1600 insect species menace stored product commodities during their production, transportation, processing, storage and marketing [1]. Species from this ever-growing list produce severe challenges to food production and storage worldwide, while developing regions are especially vulnerable [2]. In this paper we focused on the seed beetle, *Acanthoscelides obtectus* (Say), (Coleoptera: Chrysomelidae: Bruchinae), a cosmopolitan pest of stored legumes that primarily utilizes the common bean (*Phaseolus vulgaris* L.). Some studies have suggested that the beans’ annual yields can suffer a loss of 40% if infected storages are untreated [3,4]. These losses go far above the recommended economic threshold of 4% [5,6]. The total cost could be even higher if damage to all other legume species that *A. obtectus* can utilize is taken into account [7].

With estimates of around one million species, phytophagous insects are undoubtedly the most speciose group of animals [8,9]. The most conspicuous characteristic of these insects is the fact that the majority of species are host specialists, i.e., they use only one or several host plants in their diets [10]. For example, some estimates suggest that more than 90% of phytophagous insects feed on plants classified in less than three different plant families [11], implying that a host plant specialization strategy in insects has some selective advantages compared to other feeding strategies (e.g., parasitism, predatory etc.). Evolutionary branches with this strategy have high specialization and speciation rates [12,13], and such a trend is also evident in most pest species [14]. Corresponding to the “jack of all trades master of none” hypothesis, only specialists can be efficient enough in handling plants’ defenses and successful detoxification of their chemical components (for the review of the idea and modern interpretation see [15]). However, one should be very cautious with this seductive specialist-generalist dichotomy as a general paradigm [15]. According to the oscillation hypothesis, a specialist can relatively easily expand its host range, exploit alternative food sources and then specialize on a novel host plant [16,17]. Such an endeavor imposes challenges to diverse aspects of insects’ behavior, physiology, and life history strategies [18]. Thus, the question of utmost practical significance is to understand mechanisms that allow insects to expand or switch to another host plant, as well as to explain the ways in which insects become capable to sustain their populations on a novel host plant.

Survival and reproduction, the pillars of fitness along with other life history traits, can be directly translated to demographic properties of a population [19,20,21]. Designed for fundamental ecological research [22], these parameters are frequently used in assessing pest potential to invade other host plants [23,24,25,26] or plant varieties [27,28,29,30,31,32,33,34]. In the field of pest science, the adequate statistical testing and correct interpretation of abovementioned parameters is of paramount importance. Here, we used the recommended jackknife resampling procedure for calculating population parameters from life tables [35,36]. Such an approach in studying population dynamics offers a reliable and adequate statistical framework, improves the power of the analysis and makes the results more valuable.

In this paper we estimated the invading potential and assessed the population dynamics of seed beetle *Acanthoscelides obtectus* during an experimentally induced host-shift. In order to be effective in pest management strategies and to provide a swift reaction in case of infestation, insects’ population growth parameters on different host plants should be used. Additionally, such data can be used to anticipate the invading potential of a pest species on other host plants. Our experimental protocol simulates the host-shift process. Such an approach allows us to address different questions on how each specific life history trait contributes to the short- and long-term changes of population parameters. We wanted to determine if beetles reared on their optimal host change their oviposition behavior when placed on an alternative host bean species in no-choice experiments. Such a scenario simulates the first phase of potential host shifts in storages. This allows us to understand to what extent oviposition behavior affects population parameters. Next, we looked into the potential for *A. obtectus* to successfully invade and sustain its population on alternative host plants by identifying changes in life history strategies along the way. Finally, we tested how populations shifted to alternative host plant react if offered with once optimal, common bean seeds. This opens an opportunity to inspect the level of insect specialization on alternative host plants and estimate their potential to persist in storages with frequent fluctuation of stored product commodities.

The evolutionary history of *Acanthoscelides obtectus* has been very dynamic. Recent analysis on several mitochondrial genes (12s rRNA, 16s rRNA, COI) unambiguously confirmed the place of origin of *A. obtectus* in Central America [37,38] and also recognized that both pre- and post-Columbian range expansions played important roles in shaping current, worldwide distribution of this insect [39]. Additionally, the evolution of multivoltinism (i.e., the ability to produce multiple generations per year) is another important characteristic that enabled *A. obtectus* to expand the range and be highly competitive when invading stored seeds [37,40].

Several reasons qualify this holometabolic insect to be a suitable model species in empirical testing of various physiological [41,42,43], behavioral [44,45,46,47] and evolutionary hypotheses [48,49,50,51,52,53,54]. First, larvae are well adapted to dry seeds and adults are facultative aphagous (i.e., they rely only on metabolic water and resources acquired during larval development). Additionally, larval development and pupation last approximately 30 days, and adults are ready to reproduce within two hours upon emergence. Second, *A. obtectus* has a characteristic oviposition behavior, that is, females usually do not attach their eggs onto a surface of beans. This enables easy manipulation and transfer of eggs to other host plants which is especially important when studying population dynamics during the host-range expansion. Additionally, larvae are motile and could search for seeds into which they could burrow. Finally, conditions in the laboratory often resemble conditions that could be found in storages (stable temperature and humidity levels) so the long-term evolutionary experiments could be meticulously designed.

## 2. Materials and Methods

### 2.1. Laboratory Populations

We used 12 laboratory populations of *A. obtectus* reared on three host plants. Four populations were maintained on optimal—common bean seeds, *Phaseolus vulgaris* L. (Fabaceae), (hereafter referred to as ‘*Phaseolus’* or P populations—P selection regime); the remaining eight populations were maintained on the less preferable host plant: Four populations on chickpea seeds, *Cicer arietinum* L. (Fabaceae), (hereafter referred to as ‘*Cicer’* or C populations—C selection regime) and four on mung bean seeds, *Vigna radiata* L. (Fabaceae), (hereafter referred to as ‘*Mung’* or M populations—M selection regime). Prior to the experiment, replicate P and C populations evolved on common bean and chickpea seeds, respectively, for 48 generations, while M populations were selected on mung bean for 15 generations. All laboratory populations originated from the same ancestral ‘*Base*’ population collected from common beans. ‘*Base*’ population was established more than 30 years ago and reared on common beans in the laboratory ever since [52]. Within each population, at least 300 randomly sampled individuals contributed to the next generation limiting the severe effects of inbreeding. In order to decrease the effects of differences in host seed sizes, each generation was provided with the same amount of host plant seeds (approximately 150 g) in a clean glass jar, that is, proportionally more chickpea and mung bean seeds were presented to beetles in the C and M regimes than common bean seeds in the P regime.

During the experiment, insects were kept in the dark incubator set at 30 ± 1 °C. No food or water was offered to adult individuals. Chemically untreated, organic seeds were used during the course of experiment and for the maintenance of selection lines. All seeds were frozen prior to use to avoid any possible contamination.

### 2.2. Experimental Design

**Figure 1:**
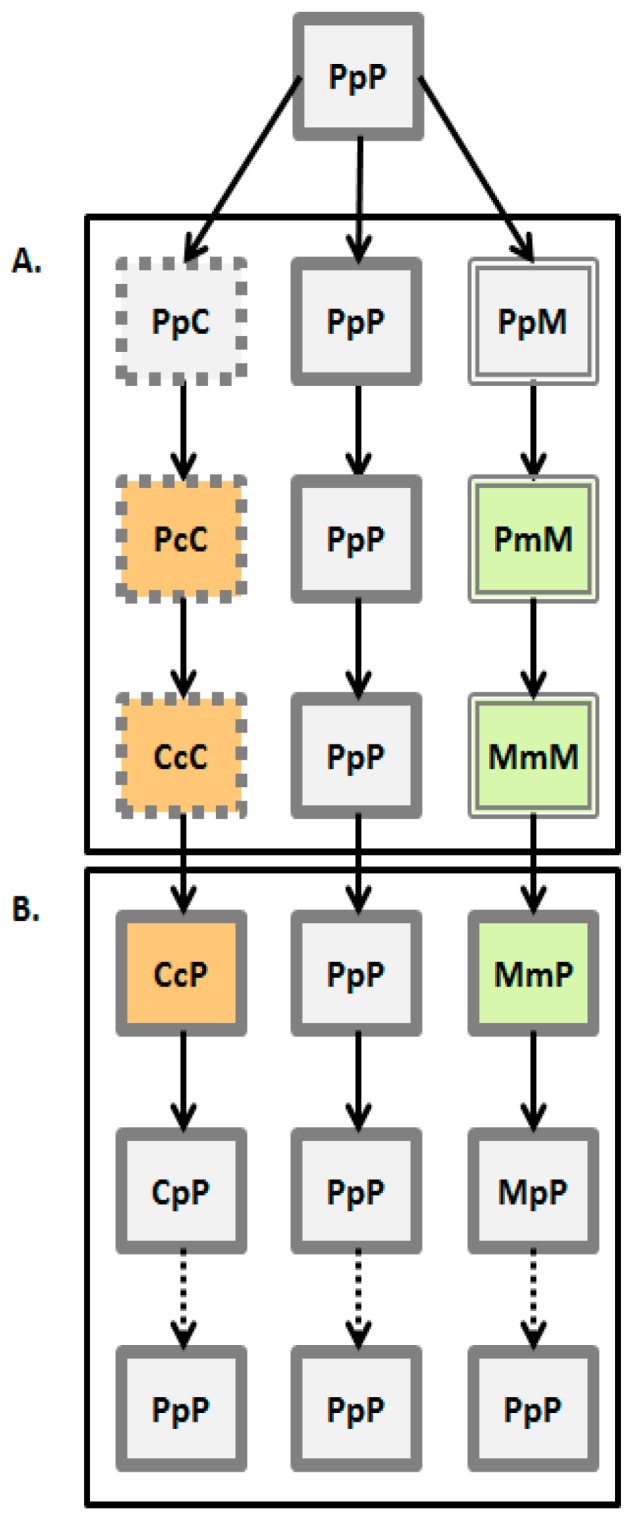
Scheme of the experimental design. Each experimental group had four replicate populations. First letter in the group name indicates the selection regime, second and third letter indicate rearing and offered ovipositing host plant, respectively (P—common bean; C—chickpea; M—mung bean). (**A**) Steps of the host-shift process (the arrows represent the path of switching to another host or remaining on the original one through time). Change of the host plant during oviposition is the initial step of the host-shift process (one generation in the experiment). Females reared on common bean (PpP group—colored in light grey and framed with solid lines) are allowed to deposit eggs on chickpea (PpC group—framed with punctuated lines) or mung bean seeds (PpM group—framed with double lines). In the next step of the host-shift, insects have to complete their development on novel chickpea (PcC group—colored in light orange) or mung bean seeds (PmM group—colored in light green)—one additional generation in the experiment. Finally, host-shift can last for many generations (48 generations in the CcC groups, and 15 generations in the MmM groups). (**B**) Steps of the reversal to common bean: Oviposition (from CcC groups selected for 48 generations on chickpea to common bean in a single generation—CcP groups, and from MmM groups selected for 15 generations on mung beans to common bean in a single generation—MmP groups) and development (CcP beetles developed on common bean for one generation—CpP, and MmP beetles developed on common bean for one generation—MpP groups). In theory, populations can return on previously common host and again evolve on it (dashed arrows, PpP group).

The experimental design is summarized in Figure 1. This experimental approach simulates several steps of the host-shift process. All beetles in this study were sub-cultured on common bean, P, using beetles from our 30-year old laboratory colony, and then reared in controlled sub-cultures on P for 48, C for 48 generations and M for 15 generations. Thus, each subculture (population) was presumed to have been exposed to some level of selection over time to adapt to either C or M. The 3-letter code of each experimental group designates the series of forced host rearing. The first uppercase letter represents the source of adult subculture selected whether from the C, P or M populations. The lower case letter, whether c, p or m, in the second position is the bean species the parent beetle was forced to infest in controlled conditions. The upper case letter in the third position is the bean species used to test performance of those beetle progeny from each of the initial transition hosts. Thus, the beetles resulting from the PpP breeding sequence should be considered the “experimental control” for this study, as these are the beetles exposed only to the original ancestral host at all forced infestations. The final progeny from the PpP crosses are then expected to have the highest values of finite rate of population increase (high fecundity rates, fast development time, high egg to adult survival).

In the first step, insects reared on the optimal host plant—bean seeds (PpP experimental group had to deposit their eggs either on chickpea or mung bean seeds (PpC and PpM groups, respectively). For this step we have paired newly hatched individuals (one female and one male per 35 mm Petri dish), measured their body mass and made daily records of their fecundity and lifespan. This allowed us to calculate early fecundity (number of deposited eggs in the first two days of life) and total fecundity on host plants. In the next phase of the host-shift offspring continues to be exposed to the novel host. In such case, both egg oviposition and larval development have to be completed on a novel host, the so called “short-term” exposure (PcC and PmM groups). In order to do so, newly hatched individuals from P populations were placed in a Petri dish with three seeds to stimulate oviposition. After 24 h, laid eggs were counted and transferred to dishes with seeds specific to P selection regime (i.e., common beans—Pp) or alternative hosts (i.e., chickpeas and mung beans—Pc and Pm). After approximately 30 days, hatching started and the number of emerged adults was recorded daily for each experimental group. This procedure was used to collect data on egg-to-adult viability and developmental time. Finally, continuous exposure to new host plant constitutes the “long-term” host-shift (CcC and MmM groups) (Figure 1A). We have applied the same experimental procedure on C and M populations in order to investigate what happens when they have to oviposit (CcP and MmP groups) and develop (CpP and MpP groups) on once optimal host plant—common bean seeds (Figure 1B).

### 2.3. Statistical Procedures

Pre-adult (egg-to-adult viability, developmental time and body mass) and adult (lifespan, early and total fecundity) life history traits were analyzed using the mix-model ANOVA models with Type III sum of squares and Satterthwaite’s approximation of denominator synthesis (SAS 9.3, Cary, NC, USA; GLM procedure). For the pre-adult traits, the selection regime, rearing host and selection regime × rearing host interaction served as fixed factors, while the replicate populations nested within selection regime × rearing host interaction were treated as random factor. Arcsine square root transformation was applied to egg-to-adult viability data following an examination of the normality and homogeneity of variance assumptions for proportion data. Being that the non-parametric Kruskal–Wallis test for developmental time revealed the same results on differences between major experimental groups (chi-square 1433.49; df = 2; pr > chi-square < 0.0001), the same statistical design was performed on this trait. For the adult life history traits, the effects of selection regime, rearing host, offered host and their interactions were treated as fixed factors, while the random factor was the same as for the pre-adult traits.

We have used life history data set to construct the life tables in order to calculate the following population parameters: The net reproductive rate (R_0_), the intrinsic rate of increase (r_m_), the mean generation time (T), the doubling time (D) and the finite rate of increase (λ) [35]. This method offers jackknife variances and confidence intervals for each population parameter making pairwise comparisons between experimental groups possible using Student *t*-test that is implemented in the procedure.

## 3. Results

**Figure 2:**
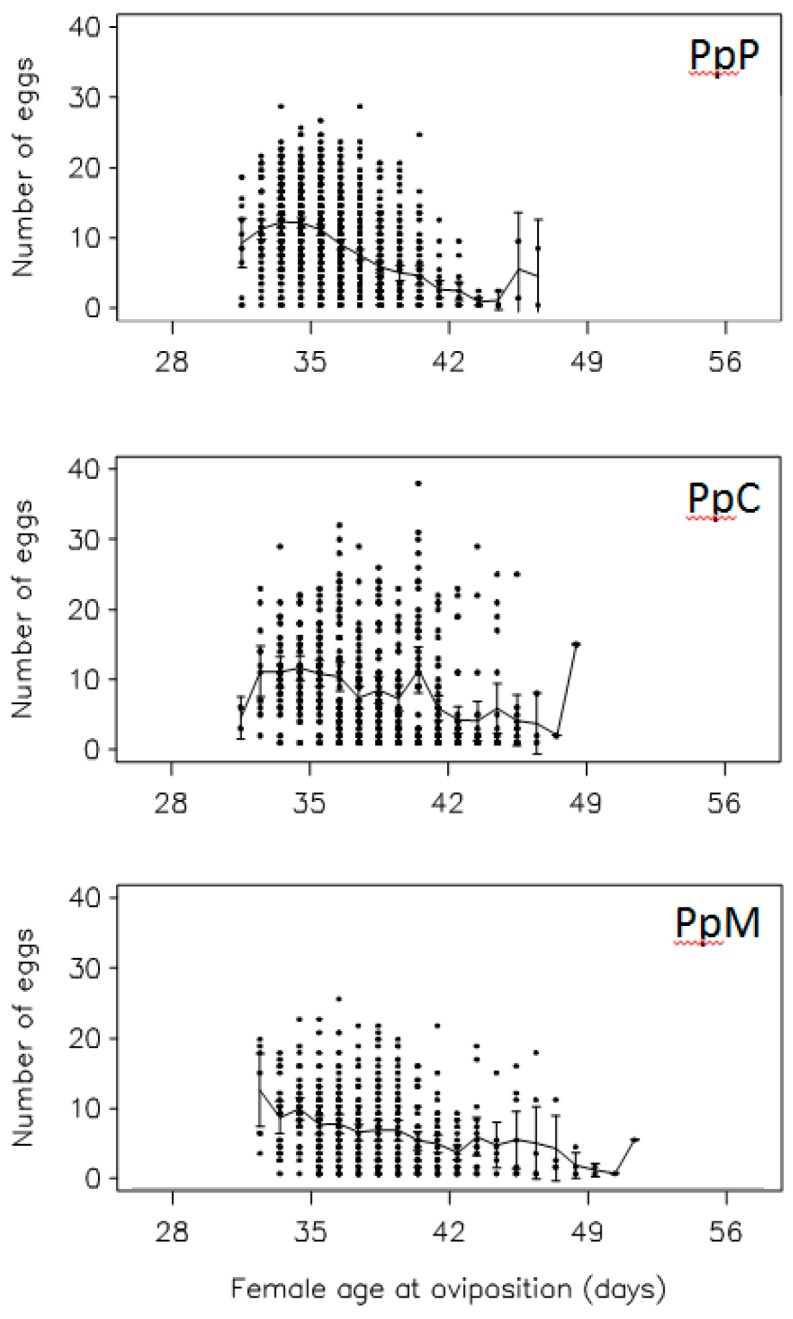
Changes in oviposition during female reproductive period: Females reared on common bean (PpP group) were allowed to deposit eggs on chickpea (PpC group) or mung bean seeds (PpM group). Raw data are shown as the individual points, with the line falling on the mean for those data with the SE of that mean.

**Figure 3:**
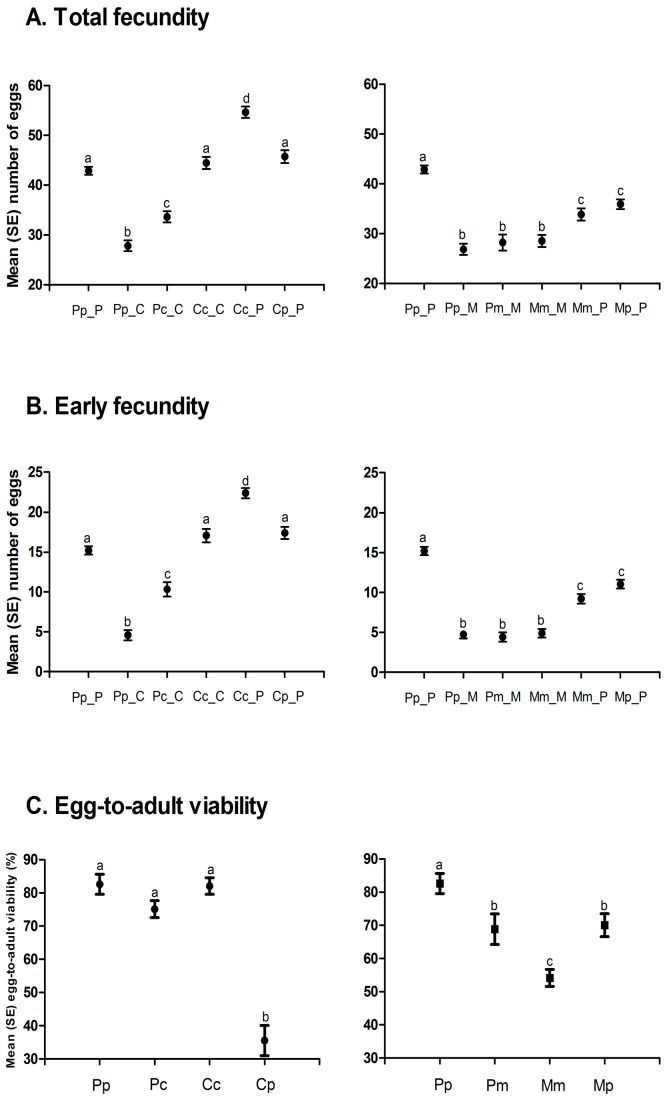
Life history traits (Mean ± SE) during experimentally induced host-shift: (**A**) Total fecundity, (**B**) early fecundity, (**C**) egg-to-adult viability. Different letters indicate statistically significant differences between groups at *p* < 0.005, Tukey’s Studentized Range (HSD) Test.

**Figure 4:**
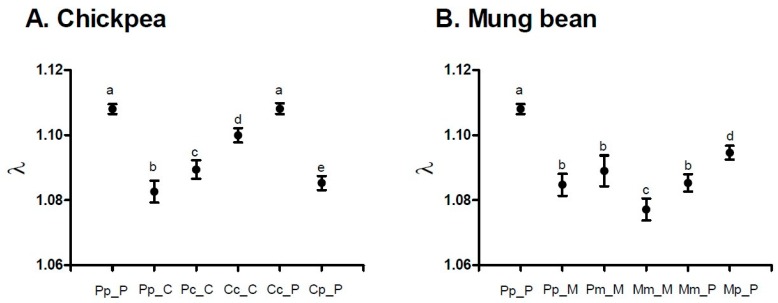
Jackknife estimates and confidence intervals of the finite rate of population increase (λ) for experimental groups during the host-shift process to: (**A**) chickpea and (**B**) mung bean seeds. Different letters indicate statistically significant differences between groups at *p* < 0.005, Student *t*-tests.

*A. obtectus* demonstrated a noticeable change in the oviposition when chickpea or mung bean seeds were offered as hosts (Figure 2). Less conspicuous oviposition peak, prolonged oviposition time and differences from a typical oviposition curve were some of the most visible elements of this change. Furthermore, differences in the oviposition schedule were accompanied with significant decrease (around 35%) of the total and the early fecundity (significant selection regime × offered host interaction in Table 1, Figure 3A,B). For example, females from PpP experimental group deposited 42.90 ± 0.75 eggs on the common bean seeds, whereas females that have been presented with chickpea (PpC group) or mung bean seeds (PpM group) had on average 27.83 ± 1.09 and 26.86 ± 1.13 eggs. Overall statistics on fecundity indicated significantly lower measures in PpC and PpM groups compared to PpP females (F = 110.64; df = 2, 654; *p* < 0.0001). Surely, such dramatic decrease of reproductive potential in novel environments left its mark on the finite rate of population increase—λ (Figure 4, Table 2). For instance, one of the highest λ values recorded in the PpP experimental group (1.10796 ± 0.00153) significantly decreased in the PpC (1.08260 ± 0.00339) and PpM (1.08470 ± 0.00336) groups (tables of statistical comparison between groups for each population parameter are presented in the supplemental material, Table S1).

After depositing the eggs, the next phase of a successful host-shift is development in a novel environment. Undoubtedly, crucial life history trait for this phase is the egg-to-adult viability (Figure 3C). Although observable, decrease in the egg-to-adult viability was not detrimental when populations selected on beans (Pp = 0.83 ± 0.03) developed on chickpea (Pc = 0.75 ± 0.02) or mung bean seeds (Pm = 0.69 ± 0.05) (F = 5.61; df = 2, 10.424; *p =* 0.0222). Additionally, compared to common bean populations (Pp = 33.01 ± 0.05), developmental time was prolonged in chickpea (Pc = 34.07 ± 0.06) and shortened in mung bean environment (Pm = 31.80 ± 0.08) (F = 20.36; df = 2, 9.2795; *p =* 0.0004) while there was no significant change of body mass after this “short-term” shift (F = 1.86; df = 2, 9.1344; *p =* 0.2097). The most interesting results were observed in the successive phases of host-shift, when there was a steady improvement of reproductive potential (increase in deposited eggs, Figure 3A), egg-to-adult viability (successful development, Figure 3C) and, consequently, finite rate of population increase (Figure 4, Table 2) in chickpea (PcC, CcC) but not in mung bean populations (PmM, MmM). Furthermore, populations reared on chickpeas for many generations almost reached the level of population growth that was observed for populations reared on the common bean, while mung bean populations have failed to do the same. For graphical representation of oviposition dynamics and population parameters of all phases of host-shift process see the supplementary Figures S1 and S2. This result clearly demonstrates that seed beetles selected on alternative host plants have changed their life history strategies.

In a constantly changing environment, encountering previously optimal host plants is a probable scenario. If populations specialized on a novel host plant are shifted back to the original host, such a situation poses a severe challenge to insect populations. Regardless of the “long-term” selection regime, our data indicate an initial improvement in population growth in the first phase of the reverse host-shift, due to increase in fecundity (Figure 3A). However, significantly lower pre-adult viability when chickpea selected populations developed on common beans (Cc = 0.82 ± 0.02 vs. Cp = 0.35 ± 0.05; F = 39.33 df = 1, 6.036; *p* = 0.0007) contrasted a situation in which mung bean selected populations displayed a significant increase in viability when developed on common beans (Mm = 0.54 ± 0.02 vs. Mp = 0.70 ± 0.03; F = 11.25; df = 1, 6.000; *p* = 0.0154) (Figure 3C). This situation indicates increased specialization rates in chickpea compared to mung bean selected populations, especially during larval development.

## 4. Discussion

Host-shift process consists of several steps that could change insects’ initial response to a new host, alter developmental, physiological and/or life history responses [18,55]. Since alterations in life history strategies, as well as adaptations at the behavioral and/or physiological levels, could easily evolve and enable pest species to utilize novel environments [7,16,17], accurate assessment of the potential host spectra is a very challenging task [56]. Undoubtedly, such range expansions could make significant damages to stored species. In order to better understand how a targeted pest species changes (evolves) during host-shift, it is essential to assess life history strategies and population dynamics on several host plants. In this paper we have used experimental evolution approach in order to inspect each host-shift phase of *A. obtectus* on two suboptimal host plants—chickpeas and mung beans.

Understanding how infestation happens must be the starting point of any pest management strategy. Undoubtedly, one of the possible scenarios is the translocation of infected bean pods or bean seeds directly from fields [57]. Although literature data on pre-harvest infestation seem to be highly variable and dependent on seasons and locations (see Table 1 in Paul et al. 2010), it seems that the short distance between farm and storage is a significant factor for higher pre-harvest infestation rates [58]. In other words, the closer the farm to storage is, the higher pre-harvest infestation rates are. However, probably the most common way of continuous infestation of a storage are remaining beetles in bins, subfloors, aeration ducts or any equipment that is used during manipulation of seeds within the storage [59]. That is why high sanitary standards should be prioritized in storages [60].

Storages frequently have different plant species from a range of geographical localities [61] providing an excellent setup for studying host range expansions. From a more commercial perspective, it is of utmost importance to know the potential of targeted pest species to adapt to new host plants. In our experiments we were able to demonstrate that the initial exposure of the seed beetles to new host plants decreased their reproductive output by more than a third. This behavioral phenomenon, in which females carefully choose the most suitable oviposition host, is not uncommon in insects and females are often under increased selection pressure to make a very precise decision on where to lay their eggs [62]. This is especially true if the larvae have little or no locomotor capacity, or the larvae are strict specialists. In many cases female choosiness delays egg deposition, which, on the other hand, increases chances that females will eventually find the most optimal host plant for larval development [48,63]. Our data indicate that reduction in the number of deposited eggs, as well as postponing of the oviposition, regardless of the identity of new host plant (i.e., chickpeas and mung bean seeds), was reflected in noticeable decrease of population growth. We have shown, however, that the pest populations could be increased in size very rapidly after developing just for a few generations on a new host. No matter how severe the population drop was, due to reduced fecundity and delayed oviposition, the seed beetles managed to complete their development within the seeds of new host plants.

Our results indicate that even though egg-to-adult viability was reduced and, consequently, resulted in a slight decrease in population growth on new hosts, seed beetles were plastic enough to secure survival of their populations on alternative seeds. One of the possible explanations for this reduction in the egg-to-adult viability could be found in different physical properties of seeds. It is known that the potential of *A. obtectus* larvae to successfully penetrate and burrow is strongly affected by the seed coat hardness. Consequently, the harder the seed coats the lower the number of larvae in endosperm is expected [64]. Since seed hardness is directly linked with moisture content of the seed [65], this could partially explain why beetles raised on mung beans have the lowest values of egg-to-adult viability. Although belonging to the Fabaceae family of plants, common bean, chickpea and mung bean seeds are very heterogeneous in their chemical compositions. For example, common bean seeds have phytochemagglutinin, lectin like α-amylase inhibitor, different types of arlequines and protease inhibitors [66,67], chickpea seeds have more than 200 secondary metabolites [68], while mung beans have naringenins, vicilins, cysteine-rich protein (VrD1 or VrCRP), vignatic acids (A and B) and para-amino-phenylalanine, chitinase [69]. Several studies suggest that phosphatases, proteins with many different functions, are partially responsible for detoxification processes during insect development [41,42]. Consequently, host-specific, short-term physiological adjustments of detoxification processes could reduce the seed beetle capacity to protect itself against xenobiotic compounds [42]. Furthermore, energy allocation trade-offs between energy demanding detoxification processes and development during the embryonic and larval stages can explain prolonged development within seeds of the new host plants. Therefore, it seems that insects, in early phases of the host-shift, could be more susceptible to commercial insecticides.

Detailed analysis of life history strategies and population parameters demonstrated host specific responses during transgenerational acceptance of the two new host plants. Our data indicated that chickpea populations were reaching levels of population growth very similar to the ones of the most optimal host plant (i.e., common bean). A steady increase in reproductive output was the main reason for this increase. Previous work on chickpea adapted seed beetles has also identified significant changes in their life strategies and reproductive behavior (e.g., lack of assortative mating patterns and changes in chemical signaling used for communication) [46]. Furthermore, low values of egg-to-adult viability, when a common bean is developing host, indicated a substantial level of host specialization on chickpea. These results are in concord with the growing body of evidence that life history evolution in species interactions can be very fast and highly dynamic, as was shown, for example, on another bruchid beetle *Callosobruchus maculatus* [70,71]. On the other side, seed beetles had much more difficulties in adjusting to mung bean seeds and their population growth on this host plant was limited. Relatively poor performance of mung bean populations, and quick recovery when placed again on common beans, could be partially explained by fewer generations of selection on this host. Nevertheless, it cannot be excluded that unique chemical signatures of mung beans could be important for increased vulnerability in this insect species, setting the limit for long-term host expansion. Our results indicate different dynamics of adapting of *A. obtectus* to diverse host plants and possibly the need for specific protection protocols. Assessing the most crucial changes in life histories, which influence a decrease in the ability of insect populations to survive and maintain on a new host plant, could be very useful for various techniques of pest reduction.

## 5. Conclusions

- Seed beetles (*Acanthoscelides obtectus*) have significant potential to colonize and maintain stable populations on several stored products from the Fabaceae plant family.
- Changes in the oviposition and decrease in reproductive output mark seed beetle populations when chickpea or mung bean seeds were offered as hosts during oviposition.
- Seed beetles selected on chickpea and mung bean seeds have changed life history strategies compared to common bean populations.
- Different legume products could have specific management protocols and ways to protect against seed beetles.

## Figures and Tables

**Table 1:** Mixed model ANOVA. Selection regime (S), rearing host (R) and offered host (O) represent the fixed factors, while populations nested within S × R interaction is the random factor. Shown are *F* values and statistical significance for: (**A**) Preadult life history traits (egg-to-adult viability, developmental time and body mass) and (**B**) adult life history traits (life span, early and total fecundity).

**A**	**Egg-to-Adult Viability**		**Developmental Time**		**Body Mass**	
	**F Value _(df)_**	***p***	**F Value _(df)_**	***p***	**F Value _(df)_**	***p***
Selection regime (S)	15.80 _(2, 25.43)_	<0.0001	36.63 _(2, 23.174)_	<0.0001	13.41 _(2, 21.374)_	0.0002
Rearing host (R)	11.53 _(2, 24.479)_	0.0003	25.25 _(2, 22.921)_	<0.0001	3.19 _(2, 21.483)_	0.0612
S × R	27.98 _(2, 22.813)_	<0.0001	2.99 _(2, 22.571)_	0.0704	0.27 _(2, 21.236)_	0.7672
Populations (S × R)	1.09_(21, 109)_	0.3713	10.76 _(21, 6662)_	<0.0001	12.68 _(21, 2647)_	<0.0001
**B**	**Life Span**		**Early Fecundity**		**Total Fecundity**	
	**F Value _(df)_**	***p***	**F Value _(df)_**	***p***	**F Value _(df)_**	***p***
Selection regime (S)	8.14 _(2, 22.646)_	0.0022	5.46 _(2, 21954)_	0.0119	23.48 _(2, 23.707)_	<0.0001
Rearing host (R)	5.85 _(2, 22879)_	0.0088	15.83 _(2, 22.086)_	<0.0001	10.67 _(2, 24.104)_	0.0005
Offered host (O)	249.83 _(2, 2637)_	<0.0001	217.79 _(2, 2584)_	<0.0001	148.37 _(2, 2642)_	<0.0001
S × R	0.29 _(2, 22.01)_	0.7482	0.46 _(2, 21.587)_	0.6386	0.13 _(2, 22.658)_	0.8767
S × O	25.59 _(2, 2637)_	<0.0001	29.29 _(2, 2584)_	<0.0001	17.65 _(2, 2642)_	<0.0001
R × O	5.02 _(2, 2637)_	0.0066	6.12 _(2, 2584)_	0.0022	5.03 _(2, 2642)_	0.0066
S × R × O	2.31 _(2, 2637)_	0.0993	1.27 _(2, 2584)_	0.2813	2.33 _(2, 2642)_	0.0974
Populations (S × R)	5.75 _(21, 2637)_	<0.0001	9.77 _(21, 2584)_	<0.0001	3.53 _(21, 2642)_	<0.0001

**Table 2:** True calculations, jackknife estimates and 95% CL for population parameters: The net reproductive rate (R_0_), the intrinsic rate of increase (r_m_), the mean generation time (T), the doubling time (D) and the finite rate of increase (λ) for each experimental group.

Experimental Group	Population Parameters				
	True Calculation Jackknife Estimate 95 % CL				
	R_0_	r_m_	T	D	λ
PpP	21.2539	0.10251	29.8156	6.76143	1.10795
	21.2539	0.10252	29.8153	6.76076	1.10796
	20.4982–22.0095	0.10114–0.10390	29.6675–29.9632	6.66979–6.85172	1.10643–1.10949
PpC	12.1634	0.07934	31.4897	8.73627	1.08257
	12.1634	0.07937	31.4855	8.72965	1.08260
	11.2084–13.1185	0.07624–0.08250	31.0955–31.8754	8.38442–9.07489	1.07921–1.08599
PcC	14.5630	0.08560	31.2915	8.09774	1.08937
	14.5667	0.08562	31.2915	8.09381	1.08939
	13.6109–15.5225	0.08301–0.08823	30.9410–31.6419	7.84689–8.34074	1.08655–1.09223
CcC	19.5765	0.095238	31.2306	7.27808	1.09992
	19.5765	0.09525	31.2304	7.27625	1.09993
	18.4509–20.7022	0.09326–0.09725	31.0281–31.4326	7.12380–7.42871	1.09774–1.10213
PpM	13.0655	0.081276	31.6204	8.52833	1.08467
	13.0655	0.08131	31.6177	8.52206	1.08470
	11.9754–14.1557	0.07821–0.08440	31.2430–31.9925	8.19685–8.84726	1.08134–1.08806
PmM	12.5430	0.085195	29.6868	8.13602	1.08893
	12.5430	0.08525	29.6832	8.12493	1.08899
	11.1002–13.9857	0.08092–0.08959	29.3225–30.0440	7.71044–8.53942	1.08427–1.09371
MmM	8.85831	0.074272	29.3699	9.33258	1.07710
	8.8583	0.07430	29.3687	9.32418	1.07713
	8.0947–9.6219	0.07113–0.07748	29.0509–29.6865	8.92493–9.72344	1.07371–1.08055
CcP	24.1158	0.10263	31.0145	6.75416	1.10808
	24.1158	0.10263	31.0145	6.75328	1.10808
	23.0292–25.2024	0.10111–0.10416	30.8560–31.1731	6.65308–6.85348	1.10640–1.10977
CpP	12.4583	0.081803	30.8348	8.47333	1.08524
	12.4598	0.08182	30.8353	8.47052	1.08526
	11.7474–13.1723	0.07983–0.08381	30.6382–31.0325	8.26446–8.67658	1.08310–1.08742
MmP	10.5369	0.081828	28.7783	8.47073	1.08527
	10.5369	0.08185	28.7786	8.46681	1.08529
	9.8103–11.2635	0.07938–0.08432	28.5039–29.0533	8.21118–8.72243	1.08261–1.08797
MpP	13.9854	0.090364	29.1933	7.67064	1.09457
	13.9854	0.09037	29.1936	7.66884	1.09458
	13.2307–14.7401	0.08843–0.09231	28.9401–29.4472	7.50415–7.83354	1.09246–1.09671

## Supplementary Materials

The following are available online at https://www.mdpi.com/2075-4450/10/6/153/s1, Table S1: Student *t*-test between group comparisons of R0, rm, T, D and λ population parameters. Figure S1: Oviposition during experimentally induced host-shift. Figure S2: Graphical plotting of population parameters (jackknife estimates) during experimentally induced host-shift.
