# Supplementary material for: Potential for Acanthoscelides obtectus to Adapt to New Hosts Seen in Laboratory Selection Experiments
Source: Insects. 2019 May 29;10(6):153. doi: 10.3390/insects10060153 (PMC6627625; doi:10.3390/insects10060153)

Figure S1. Oviposition during experimentally induced host-shift.

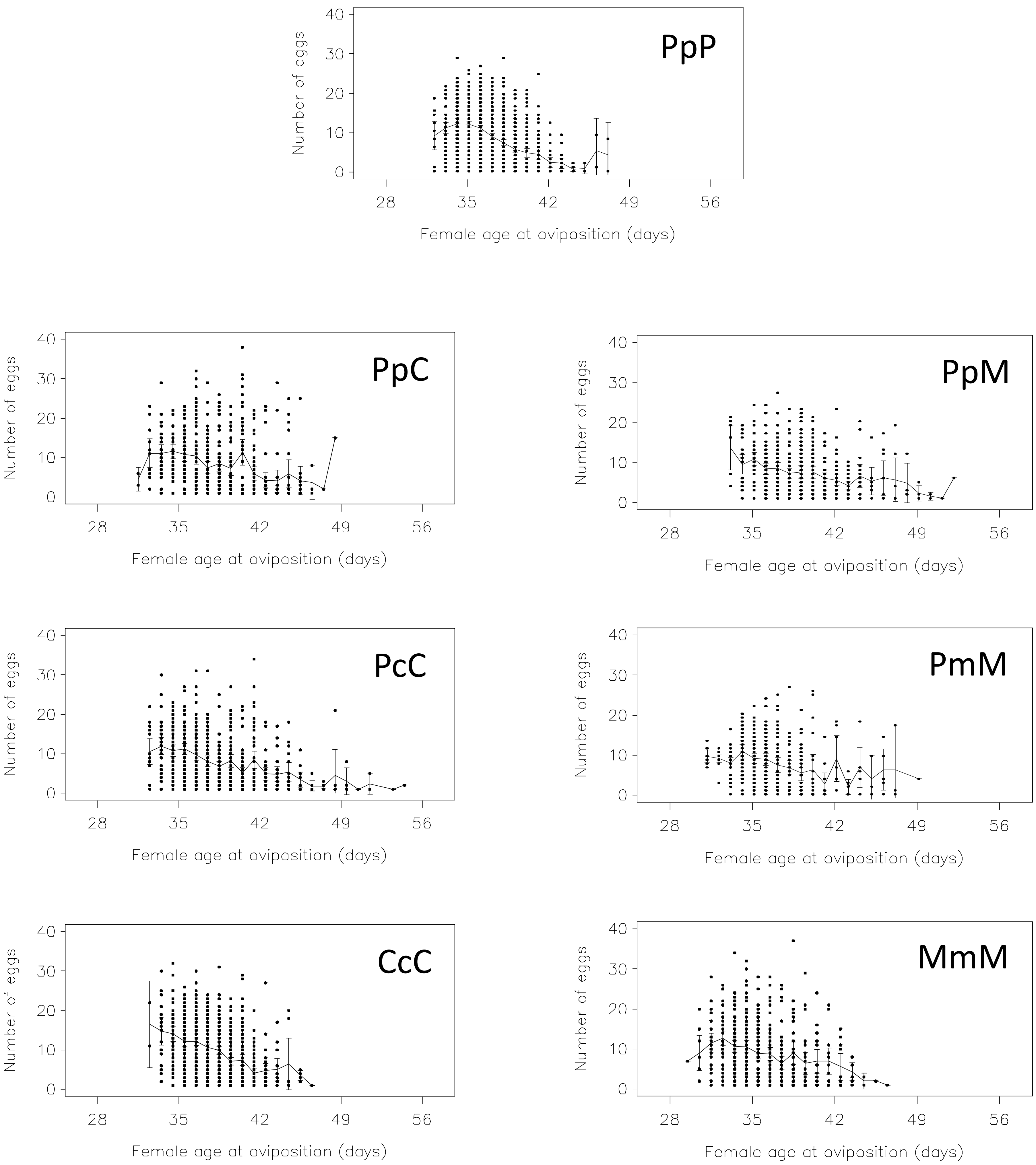

Figure S1 cont. Oviposition during experimentally induced host-shift.

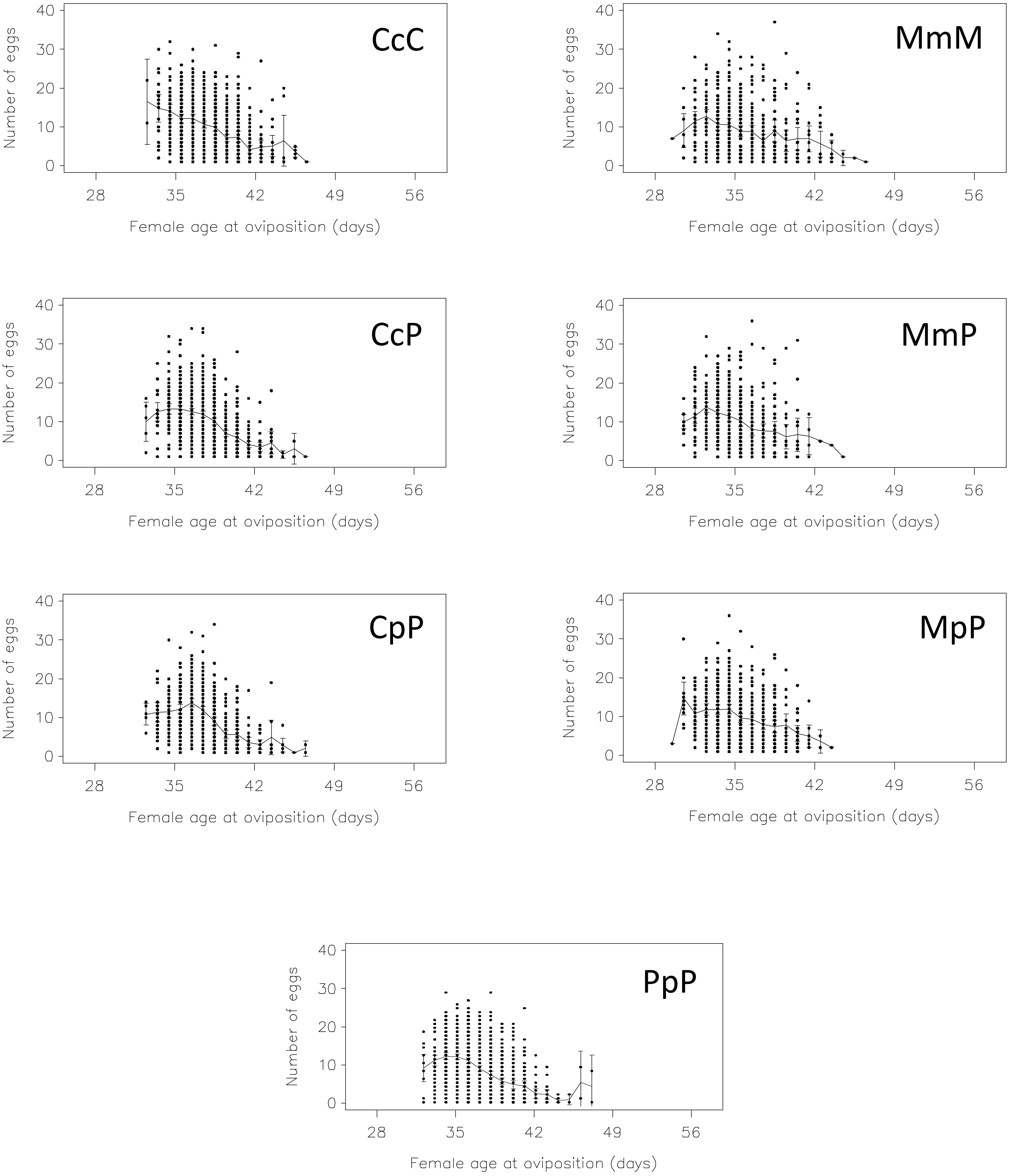

Supplement: Supplementary file 1 [file insects-10-00153-s001.zip › Figure S1..pdf]
