# Supplementary material for: Potential for Acanthoscelides obtectus to Adapt to New Hosts Seen in Laboratory Selection Experiments
Source: Insects. 2019 May 29;10(6):153. doi: 10.3390/insects10060153 (PMC6627625; doi:10.3390/insects10060153)
Supplement: Supplementary file 1 [file insects-10-00153-s001.zip › Table S1..pdf]

Table S1. Student t-test between group comparisons of R0, rm, T, D and λ population parameters.

λ	CcC	CcP	CpP	MmM	MmP	MpP	PcC	PmM	PpC	PpM	PpP
CcC	/										
CcP	***	/									
CpP	***	***	/								
MmM	***	***	***	/							
MmP	***	***	ns	***	/						
MpP	***	***	***	***	***	/					
PcC	***	***	*	***	*	**	/				
PmM	***	***	ns	***	ns	*	ns	/			
PpC	***	***	ns	*	ns	***	**	*	/		
PpM	***	***	ns	**	ns	***	*	ns	ns	/	
PpP	***	ns	***	***	***	***	***	***	***	***	/

Rm	CcC	CcP	CpP	MmM	MmP	MpP	PcC	PmM	PpC	PpM	PpP
CcC	/										
CcP	***	/									
CpP	***	***	/								
MmM	***	***	***	/							
MmP	***	***	ns	***	/						
MpP	***	***	***	***	***	/					
PcC	***	***	*	***	*	**	/				
PmM	***	***	ns	***	ns	*	ns	/			
PpC	***	***	ns	*	ns	***	**	*	/		
PpM	***	***	ns	**	ns	***	*	ns	ns	/	
PpP	***	ns	***	***	***	***	***	***	***	***	/

D	CcC	CcP	CpP	MmM	MmP	MpP	PcC	PmM	PpC	PpM	PpP
CcC	/										
CcP	***	/									
CpP	***	***	/								
MmM	***	***	***	/							
MmP	***	***	ns	***	/						
MpP	***	***	***	***	***	/					
PcC	***	***	*	***	*	**	/				
PmM	***	***	ns	***	ns	*	ns	/			
PpC	***	***	ns	*	ns	***	**	*	/		
PpM	***	***	ns	**	ns	***	*	ns	ns	/	
PpP	***	ns	***	***	***	***	***	***	***	***	/

R0	CcC	CcP	CpP	MmM	MmP	MpP	PcC	PmM	PpC	PpM	PpP
CcC	/										
CcP	***	/									
CpP	***	***	/								
MmM	***	***	***	/							
MmP	***	***	***	**	/						
MpP	***	***	**	***	***	/					
PcC	***	***	***	***	***	ns	/				
PmM	***	***	ns	***	*	ns	*	/			
PpC	***	***	ns	***	**	**	***	ns	/		
PpM	***	***	ns	***	***	ns	*	ns	ns	/	
PpP	*	***	***	***	***	***	***	***	***	***	/

T	CcC	CcP	CpP	MmM	MmP	MpP	PcC	PmM	PpC	PpM	PpP
CcC	/										
CcP	ns	/									
CpP	**	ns	/								
MmM	***	***	***	/							
MmP	***	***	***	**	/						
MpP	***	***	***	ns	*	/					
PcC	ns	ns	*	***	***	***	/				
PmM	***	***	***	ns	***	*	***	/			
PpC	ns	*	**	***	***	***	ns	***	/		
PpM	ns	**	***	***	***	***	ns	***	ns	/	
PpP	***	***	***	*	***	***	***	ns	***	***	/
